# Fusaricidin Produced by *Paenibacillus polymyxa* WLY78 Induces Systemic Resistance against Fusarium Wilt of Cucumber

**DOI:** 10.3390/ijms20205240

**Published:** 2019-10-22

**Authors:** Yunlong Li, Sanfeng Chen

**Affiliations:** State Key Laboratory of Agrobiotechnology and College of Biological Sciences, China Agricultural University, Beijing 100094, China; lyl4840361@cau.edu.cn

**Keywords:** biocontrol, gene disruption, *Paenibacillus polymyxa*, induced systemic resistance

## Abstract

Cucumber is an important vegetable crop in China. Fusarium wilt is a soil-borne disease that can significantly reduce cucumber yields. *Paenibacillus polymyxa* WLY78 can strongly inhibit *Fusarium oxysporum* f. sp. *Cucumerium*, which causes Fusarium wilt disease. In this study, we screened the genome of WLY78 and found eight potential antibiotic biosynthesis gene clusters. Mutation analysis showed that among the eight clusters, the fusaricidin synthesis (*fus*) gene cluster is involved in inhibiting the *Fusarium* genus, *Verticillium albo-atrum*, *Monilia persoon*, *Alternaria mali*, *Botrytis cinereal*, and *Aspergillus niger*. Further mutation analysis revealed that with the exception of *fusTE*, the seven genes *fusG*, *fusF*, *fusE*, *fusD*, *fusC*, *fusB*, and *fusA* within the *fus* cluster were all involved in inhibiting fungi. This is the first time that demonstrated that *fusTE* was not essential. We first report the inhibitory mode of fusaricidin to inhibit spore germination and disrupt hyphal membranes. A biocontrol assay demonstrated that fusaricidin played a major role in controlling Fusarium wilt disease. Additionally, qRT-PCR demonstrated that fusaricidin could induce systemic resistance via salicylic acid (SA) signal against Fusarium wilt of cucumber. WLY78 is the first reported strain to both produce fusaricidin and fix nitrogen. Therefore, our results demonstrate that WLY78 will have great potential as a biocontrol agent in agriculture.

## 1. Introduction

Cucumber is an important vegetable crop in China. *Fusarium oxysporum* f. sp. *cucumerium* is the causal agent of the Fusarium wilt of cucumber, a soil-borne disease that can significantly reduce cucumber yields [1]. To suppress this disease, various pesticides are widely used and cause more problems due to the detrimental effects of pesticide residues on human health. Additionally, the overuse of nitrogen fertilizer in agriculture leads to soil, water, and air pollution [2]. Therefore, the application of biocontrol agents that could both suppress disease and fix nitrogen is considered to be a promising management strategy.

*Paenibacillus polymyxa* strains offer a biological solution to suppress plant diseases due to their ability to produce some compounds that inhibit plant pathogens and are listed as commercial biocontrol agents by the United States Environment Protection Agency [3,4]. *P. polymyxa* strains have been used as biocontrol agents in controlling crown rot disease, hairy root disease, grapevine aerial disease, Phytophthora blight of pepper, Fusarium wilt of watermelon, and blackleg disease of canola [5,6,7,8,9,10].

Several mechanisms have been attributed to biocontrol agents for inhibiting plant diseases: the synthesis of antibiotics, root-colonization, and the induction of plant resistance [11]. A wide variety of bacteria, especially members of the *Bacillus* and *Paenibacillus* genera, can produce antimicrobial lipopeptides [12,13,14]. These lipopeptides include the polymyxins, polypeptins, iturins, surfactins, fusaricidins, fengycins, tridecaptins, and others [15]. Bacterial lipopeptides are secondary metabolites, generally produced by non-ribosomal peptide synthetase, and often exhibit broad-spectrum antimicrobial activity [16]. 

Fusaricidins are a class of cyclic lipopeptide antibiotics produced by *P. polymyxa*, which contain a cyclic polypeptide (CP) consisting of six amino acids and a guanidinylated β-hydroxy fatty acid (GHPD) [17]. Several kinds of fusaricidins have been isolated and characterized from *P. polymyxa* strains. *P. polymyxa* strain L1129 produced a peptide antibiotic complex, named LI-F, from which five kinds of fusaricidins LI-F03, F04, F05, F07, and F08 were isolated [18]. Four kinds of fusaricidins, named as A, B, C, and D, which are also referred to as the LI-F series antibiotics, were isolated from *P. polymyxa* KT-8 and from *P. polymyxa* SQR-21 [12,19]. All kinds of fusaricidins have shown antimicrobial activities against Gram-positive bacteria and *Fusarium* genus in vitro. The possible antibacterial mechanism of fusaricidin is that the compound interacts with the cytoplasmic membranes, while the inhibitory mode of action of fusaricidin to inhibit fungi remains unclear [20]. Additionally, the likely hypothesis that fusaricidin suppresses *Fusarium* wilt of cucumber in vivo has not been rigorously proven up to now.

In addition, orfamide, a lipopeptide antibiotic produced by *Pseudomonas* spp., was reported to induce systemic resistance in rice [21]. The induced system resistance (ISR) is mediated by ethylene and usually responds to certain beneficial and non-pathogenic rhizobacteria [22]. Interestingly, three lipopeptide antibiotics (i.e. surfactin, iturin, and fengycin) produced by rhizobacteria *Bacillus* spp. were reported to induce ISR via the salicylic acid (SA) pathway, rather than the ethylene pathway [23,24,25]. It is of note that systemic acquired resistance (SAR) is dependent on SA and usually responds to pathogens. In SAR, non-expressor of pathogenesis-related genes 1 (NPR1) functions as a co-activation of SA-responsive (pathogenesis-related) *PR* genes [26], while the role of NPR1 in ISR seems to be different. It is, however, clear that both two resistances require NPR1. After pathogen challenge, SAR can be rapidly elicited by the accumulation of SA [27]. SAR can activate a set of *PR* genes coding for proteins with antimicrobial activities in plant, providing plants with long-term resistance against subsequent pathogen infections [28,29]. Previously, we reported that *P. polymyxa* WLY78 could colonize the rhizosphere of wheat, maize, and cucumber [30]. However, it is still a mystery as to whether any types of plant resistance can be induced by rhizobacteria *P. polymyxa* WLY78.

*P. polymyxa* WLY78, a nitrogen-fixer, showed strong antifungal activity against *F. oxysporum* f. sp. *cucumerium* in vitro [31,32]. In this study, to reveal what kind of compounds could be produced by *P. polymyxa* WLY78 to inhibit plant pathogens, eight potential antibiotic biosynthesis gene clusters located on the genome of *P. polymyxa* WLY78 were predicted to be candidates. Mutation analysis revealed that the fusaricidin synthesis (*fus*) gene cluster was involved in inhibiting not only the *Fusarium* genus, but also *Verticillium albo-atrum*, *Monilia persoon*, *Alternaria mali*, *Botrytis cinereal*, and *Aspergillus niger*. The previously reported *fus* gene cluster consists of eight genes (i.e. *fusG*, *fusF*, *fusE*, *fusD*, *fusC*, *fusB*, *fusA,* and *fusTE*). Further mutation analysis revealed that with the exception of the *fusTE* gene, the seven genes *fusA*, *fusB*, *fusC*, *fusD*, *fusE*, *fusF*, and *fusG* within the *fus* gene cluster were all involved in antifungal activity. This is the first time that the *fusTE* gene has been demonstrated to not be essential for fusaricidin production. Moreover, we first report that the inhibitory mode of the action of fusaricidin inhibits spore germination and disrupts the hyphal tips of fungi by causing cytoplasm leakage. Importantly, our study also revealed that fusaricidin elicits the SA-mediated system resistance for the first time. *P. polymyxa* WLY78 is also the first reported strain that could both produce seven forms of fusaricidins and fix nitrogen. Taken together, our results show that *P. polymyxa* WLY78 has great potential as a biocontrol agent in agriculture. 

## 2. Results

### 2.1. The fus Gene Cluster in P. polymyxa WLY78 Is Essential for Antifungal Activity

Initially, we examined the antifungal activities of the four *Paenibacillus* species (*P. zanthoxyli* JH29, *P. beijingensis* 1-18, *P. sabinae* T27, and *P. polymyxa* WLY78) and two *Bacillus* species (*B. amyloliquefaciens* LJ02 and *B. subtilis* 168). We found that *P. polymyxa* WLY78 showed an excellent antifungal activity against *F. oxysporum* f. sp. *cucumerium* (Figure S1). Additionally, methanol extracts from *P. polymyxa* WLY78 cells exhibited antifungal activity from −20 to 90 °C, and the heat-stable character suggests that the antifungal compound should be secondary metabolites rather than proteins or enzymes (Figure S2).

**Figure 1:**
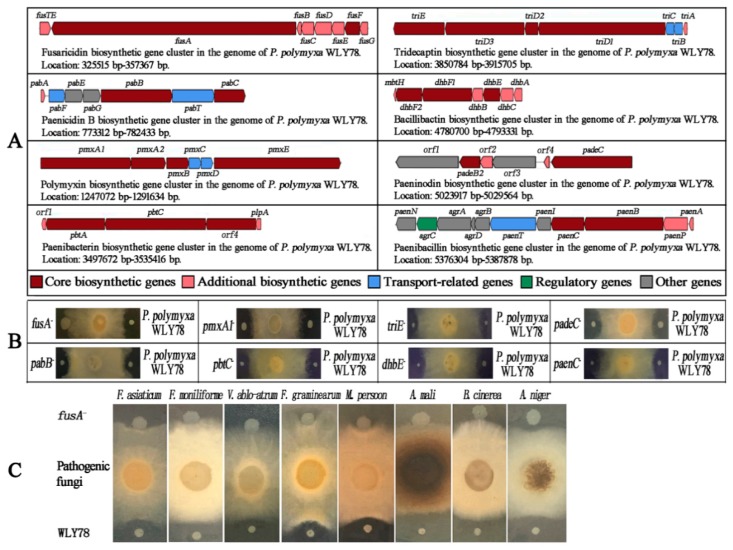
Structures of the eight gene clusters that might be involved in antibiotic production. The core biosynthetic gene indicates that the structural gene is directly involved in synthesizing the core structure of antibiotics. The additional biosynthetic gene indicates that the structural gene is involved in synthesizing the attachment group of antibiotics (**A**). Antifungal activities of each mutant (*fusA^−^*, *pabB^−^*, *pmxA1^−^*, *pbtC^−^*, *triE^−^*, *dhbE^−^*, *padeC^−^*, and *paenC^−^*) against *F. oxysporum* f. sp. *cucumerium* (**B**). Antifungal activities of the *fusA^−^* mutant against *F. asiaticum*, *F. moniliforme*, *V. albo-atrum*, *F. graminearum, M. persoon*, *A. mali*, *B. cinereal*, and *A. niger* (**C**).

Furthermore, eight gene clusters in *P. polymyxa* WLY78 that might be involved in the synthesis of the antibiotic were predicted by using antiSMASH software (Figure 1A). DNA homology analysis showed that each of the eight gene clusters exhibited 40–100% similarities with the corresponding gene clusters for antibiotic biosynthesis from *Paenibacillus* species or *Bacillus* species. For example, the fusaricidin biosynthesis (*fus*) gene cluster of *P. polymyxa* WLY78 showed 95% similarity with that of *P. polymyxa* PKB1 (GenBank: EF451155.3). The paenicidin B biosynthesis (*pab*) gene cluster of *P. polymyxa* WLY78 showed 100% similarity with that of *P. terrae* NRRL B-30644 (GenBank: KF111343.1). The polymyxin biosynthesis (*pmx*) gene cluster of *P. polymyxa* WLY78 showed 100% similarity with that of *P. polymyxa* PKB1 (GenBank: JN660148.1). The paenibacterin biosynthesis (*pbt*) gene cluster of *P. polymyxa* WLY78 showed 60% similarity with that of *Paenibacillus* sp. OSY-SE (GenBank: JX899679.1). The tridecaptin biosynthesis (*tri*) gene cluster of *P. polymyxa* WLY78 showed 100% similarity with that of *P. terrae* NRRL B-30644 (GenBank: KF111342.1). The bacillibactin biosynthesis (*dhb*) gene cluster of *P. polymyxa* WLY78 showed 53% similarity with that of *B. subtilis* subsp. *subtilis* str. 168 (GenBank: AL009126.3). The paeninodin biosynthesis (*pade*) gene cluster of *P. polymyxa* WLY78 showed 40% similarity with that of *P. dendritiformis* C454 (GenBank: AHKH01000064.1). The paenibacillin biosynthesis (*paen*) gene cluster of *P. polymyxa* WLY78 showed 100% similarity with that of *P. polymyxa* OSY-DF (GenBank: JQ728481.1). For more details, please refer to Supplementary Tables S7–S13. The high similarities (>95%) of these gene clusters indicate that *P. polymyxa* WLY78 might produce corresponding antibiotics including fusaricidin, paenicidin, polymyxin, tridecaptin, and paenibacillin. 

To identify which gene cluster might mainly be involved in inhibiting *F. oxysporum* f. sp. *cucumerium*, the core structural genes of each of the eight clusters (*fus*, *pab*, *pmx*, *pbt*, *tri*, *dhb*, *pade*, and *paen*) were inactivated via homologous recombination, yielding eight mutants including *fusA^−^*, *pabB^−^*, *pmxA1^−^*, *pbtC^−^*, *triE^−^*, *dhbE^−^*, *padeC^−^*, and *paenC^−^*, and these mutants were identified by PCR (Figure S3). Then, the antifungal abilities of these mutants against *F. oxysporum* f. sp. *cucumerium* were examined. As shown in Figure 1B, the *fusA^−^* mutant completely lost its antifungal activity, while the other mutants (*pabB^−^*, *pmxA1^−^*, *pbtC^−^*, *triE^−^*, *dhbE^−^*, *padeC^−^*, and *paenC^−^*) showed the same antifungal activities as *P. polymyxa* WLY78. In addition, the *fusA^−^* mutant lost its antifungal activity against *F. asiaticum*, *F. moniliforme*, *V. albo-atrum*, *F. graminearum*, *M. persoon*, *A. mali*, *B. cinereal,* and *A. niger* (Figure 1C). In addition, the *fusA^−^* mutant showed similar physiological and biochemical properties with wild-type *P. polymyxa* WLY78 such as the utilization of starch, l-arabinose, d-trehalose, and glycerol (Supplementary Table S6). Furthermore, the cell dry weights of *P. polymyxa* WLY78 and the *fusA^−^* mutant harvested at two days and three days of cultivation exhibited similar values (Figure S5A). The nitrogenase activities were similar in the *fusA^−^* mutant and *P. polymyxa* WLY78 (Figure S5B). These findings suggest that the *fus* gene cluster is involved in inhibiting pathogenic fungi, but does not affect the cell growth, nitrogen-fixation, physiological, or biochemical properties.

### 2.2. The Role of Each Gene within the fus Gene Cluster in Inhibition of F. oxysporum f. sp. cucumerium

The predicted *fus* cluster in *P. polymyxa* WLY78 consisted of eight open reading frames (ORFs) arranged within a 31.8 kb region in the following order: *fusG, fusF, fusE, fusD, fusC, fusB, fusA*, and *fusTE*. Of the eight genes, *fusA* is the largest gene with a length of 23.73 kb, which is responsible for synthesizing the cyclic polypeptide (CP) moiety of fusaricidin. The products of *fusG*, *fusF*, *fusE*, *fusD*, *fusc*, and *fusB* are responsible for synthesizing the lipid moiety of fusaricidin (Table 1).

**Figure 2:**
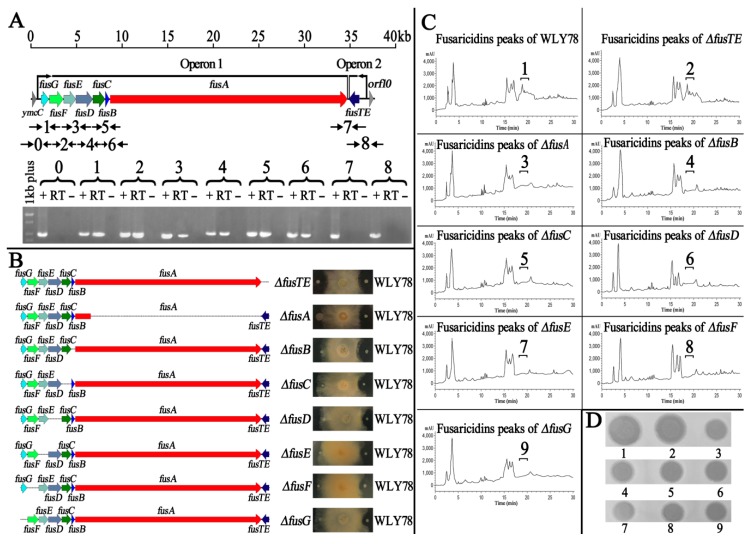
The *fusTE* is not involved in producing fusaricidins. Organization of the eight genes within the *fus* gene cluster in *P. polymyxa* WLY78 was determined by RT-PCR. The numbers on the top of the gels correspond to the product numbers drawn schematically in the outline given above. RT is the standard RT-PCR reaction; (–) is the negative control in which no reverse transcriptase was added to the RT reaction; (+) is the positive control in which genomic DNA was used as the template in the RT reaction (**A**). Antifungal activity of *fus* gene mutants (*fusA^−^*, *ΔfusB*, *ΔfusC*, *ΔfusD*, *ΔfusE*, *ΔfusF*, *ΔfusG*, and *ΔfusTE*) against *F. oxysporum* f. sp. *cucumerium* (**B**). Collection of fusaricidins extracted from wild-type WLY78 (1), *ΔfusTE* (2), *fusA^−^* (3), *ΔfusB* (4), *ΔfusC* (5), *ΔfusD* (6), *ΔfusE* (7), *ΔfusF* (8), and *ΔfusG* (9) cells, according to the elution time between 18.7 min and 20.6 min (**C**), and then each fraction was bio-assayed against *F. oxysporum* f. sp. *cucumerium* (**D**).

RT-PCR analysis indicated that the seven genes *fusG*, *fusF*, *fusE*, *fusD*, *fusC*, *fusB*, and *fusA* are organized as an operon with a length of 30.7 kb, while *fusTE* (1035 bp) is independently transcribed by its own promoter (Figure 2A).

As described above, the mutation of *fusA* within the *fus* cluster led to the loss of antifungal activities. Therefore, the other seven genes *fusG, fusF, fusE, fusD, fusC, fusB*, and *fusTE* within the *fus* cluster were individually inactivated, yielding the seven mutants: *ΔfusB*, *ΔfusC*, *ΔfusD*, *ΔfusE*, *ΔfusF*, *Δ fusG*, and *ΔfusTE*, and these mutants were identified by PCR (Supplementary Figure S4). Then, the antifungal abilities of these mutants against *F. oxysporum* f. sp. *cucumerium* were examined. As shown in Figure 2B, *ΔfusB*, *ΔfusC*, *ΔfusD*, *ΔfusE*, *ΔfusF*, and *ΔfusG* lost their inhibition effect against *F. oxysporum* f. sp. *cucumerium*, while *ΔfusTE* had the same antifungal activities as *P. polymyxa* WLY78. Taken together, our results demonstrate that *fusG, fusF, fusE, fusD, fusC, fusB*, and *fusA* are essential for inhibiting *F. oxysporum* f. sp. *cucumerium*, but *fusTE* is not. This is the first time that *fusTE* has been revealed to be not essential for inhibiting plant pathogens. Furthermore, the methanol extracts of wild-type *P. polymyxa* WLY78 and these *fus* mutants were also analyzed by HPLC. Only the extracts from wild-type WLY78 and *ΔfusTE* strains showed several *A_210_* peaks eluting between 18.7 min and 20.6 min from the reversed-phase column, and only the eluents collected from the wild-type and *ΔfusTE* showed antifungal activity against *F. oxysporum* f. sp. *cucumerium*, which was consistent with the character of fusaricidins (Figure 2C,D). All of these results demonstrate that *fusTE* is not involved in the synthesis of fusaricidins.

### 2.3. P. polymyxa WLY78 Produces Seven Forms of Fusaricidins and Fixes Nitrogen

**Figure 3:**
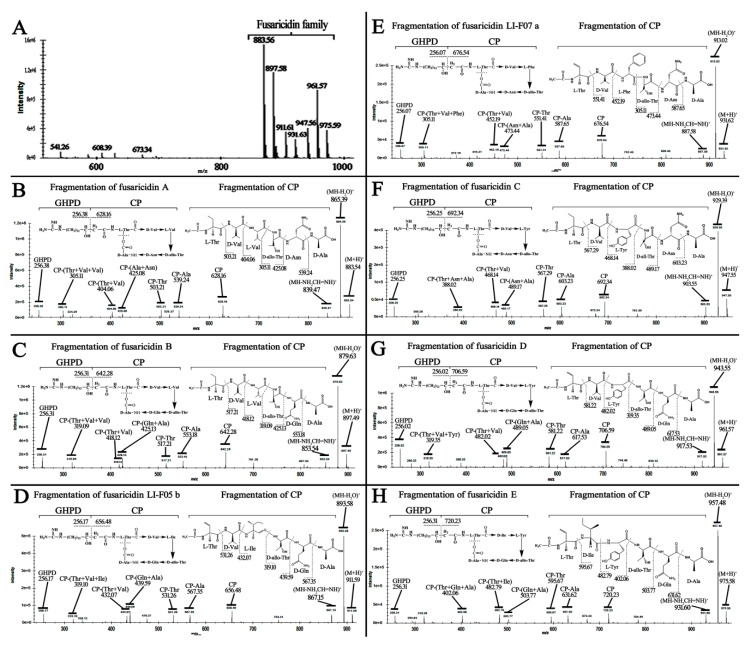
LC-MS analysis of fusaricidins extracts from *P. polymyxa* WLY78 (**A**). The fragmentation patterns of fusaricidin structures when using *m*/*z* 883.56 (**B**), 897.58 (**C**), 911.61 (**D**), 931.63 (**E**), 947.56 (**F**), 961.57 (**G**), and 975.59 (**H**) as a precursor ion through MS–MS fragment ion analysis, respectively.

Liquid chromatography-mass spectrum (LC-MS) analysis showed that *P. polymyxa* WLY78 might produce a mixture of fusaricidins, with seven forms of molecular ions including A (883 Da), B (897 Da), LI-F05b (911 Da), LI-F07a (931 Da), C (947 Da), D (961 Da), and E (975 Da) (Figure 3A). These molecular ions were respectively used as precursor ions for MS–MS fragment ion analysis to elucidate their structures. Figure 3B shows that the ion of *m*/*z* 883 was used as precursor ions, the ion of *m*/*z* 256.38 represents the 15-guanidino-3-hydroxypentadecanoic acid (GHPD) side chain, and the ion of *m*/*z* 628.16 represents the cyclic polypeptide (CP) of fusaricidin A (883.54). Furthermore, the N-terminal stepwise cleavage of CP was Thr (503.21), Val (404.06), and Val (305.11), while the C-terminal stepwise cleavage of CP was Ala (539.24) and Asn (425.08). Therefore, the fracture fragments for the ion of *m*/*z* 883 were exactly consistent with the molecular structure and fragmentation pattern of fusaricidin A. Using a similar method, the other six molecular ions of *m*/*z* 897, 911, 931, 947, 961, and 975 were confirmed as fusaricidin B, LI-F05b, LI-F07a, C, D, and E, respectively (Figure 3C–H). In addition, *P. polymyxa* WLY78 were determined to show nitrogenase activities (Figure S5B). All of these results demonstrate that *P. polymyxa* WLY78 produces seven forms of fusaricidin and fixes nitrogen.

### 2.4. Fusaricidin Inhibits Spore Germination and Hypha Growth by Causing Cytoplasm Leakage

**Figure 4:**
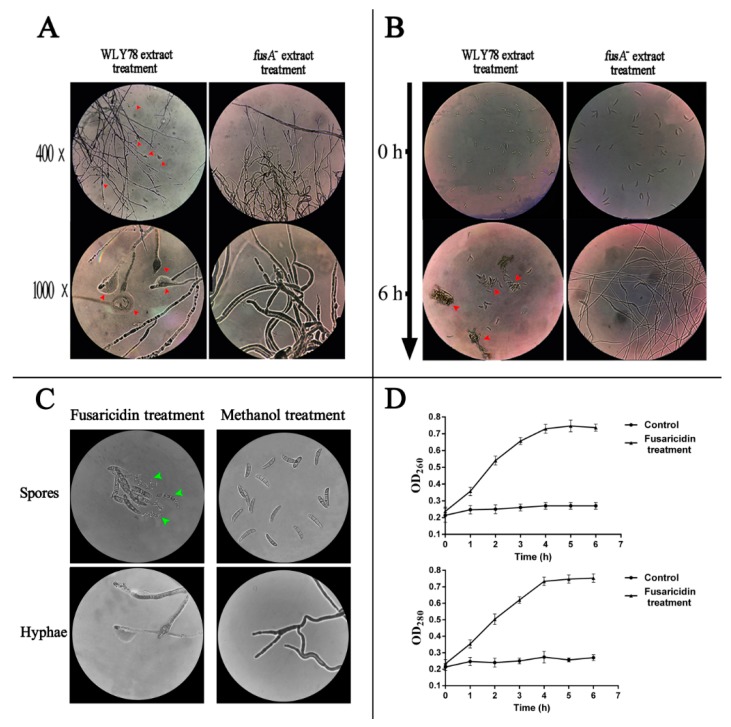
400× and 1000× micrographs from an optical microscope for hyphae treated with WLY78 and the *fusA^−^* mutant extracts (**A**). The 100× micrograph from an optical microscope for the germination of spores treated with WLY78 and the *fusA^−^* mutant extracts for six hours (**B**). 1000× micrographs from an optical microscope for spores and hyphae treated with or without fusaricidin for two hours (**C**). Leakage levels of nucleic acid (OD_260_) and proteins (OD_280_) of *F. oxysporum* f. sp. *cucumerium* spore suspensions after treatment with or without purified fusaricidin were evaluated (**D**). The red arrows indicate the vesicle structures, the cytoplasm leakage, and spore aggregation. The green arrows indicate the spores bursting into pieces.

To reveal the inhibitory mode of fusaricidin against fungi, the methanol extracts from *P. polymyxa* WLY78 and the *fusA^−^* mutant were individually added to the suspensions of *F. oxysporum* f. sp. *cucumerium* spores and hyphae. After incubation for different times at 28 °C, the spores and hyphae were observed under an optical microscope. The hyphal tips treated with the methanol extracts from *P. polymyxa* WLY78 were expanded and ruptured, while those treated with the *fusA^−^* mutant extracts grew well (Figure 4A). Initially, the spores treated with the *P. polymyxa* WLY78 and with the *fusA^−^* mutant extracts showed similar forms. However, after 6 h of incubation, the spores treated with the *P. polymyxa* WLY78 extracts aggregated, precipitated, and did not germinate, while the spores treated with the *fusA^−^* mutant extracts germinated and formed long hyphae (Figure 4B). Furthermore, the purified fusaricidin obviously accelerated this process into two hours and caused spores to burst into pieces (Figure 4C). Moreover, the effects of purified fusaricidins can be reflected in the leakage of intracellular nucleic acid and proteins by changes in OD_260_ and OD_280_, respectively (Figure 4D). It can be seen that the OD_260_ and OD_280_ of the extracellular fluids increased rapidly in the first four hours. These results reveal that the inhibitory mode of action of fusaricidin is to inhibit spore germination and hypha growth by causing cytoplasm leakage.

### 2.5. Fusaricidin Suppresses Fusarium Wilt of Cucumber In Vivo

**Figure 5:**
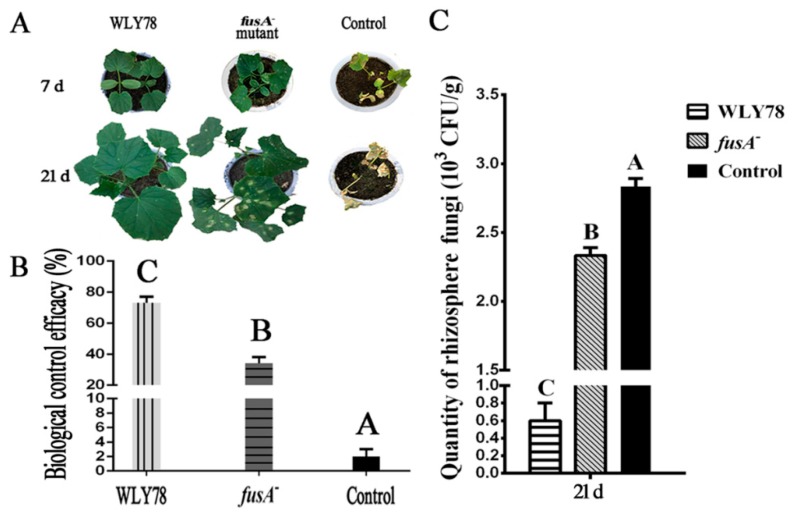
The disease symptoms of Fusarium wilt of cucumber after treatment with *P. polymyxa* WLY78, the *fusA*^−^ mutant, and water, respectively (**A**). Biological control efficacy (%) of *P. polymyxa* WLY78, the *fusA*^−^ mutant, and water against Fusarium wilt of cucumber (**B**). Quantities of *F. oxysporum* f. sp. *cucumerium* in rhizosphere after treatment with *P. polymyxa* WLY78, the *fusA*^−^ mutant, and water (**C**). Error bars indicate standard deviations among triplicates. Different letters indicate significant differences at *p* < 0.01 according to the Duncan multiple range test.

Here, the biocontrol effects of *P. polymyxa* WLY78 and the *fusA^−^* mutant on Fusarium wilt of cucumber were comparatively analyzed. The cucumber seedlings at seven days after infection with *F. oxysporum* f. sp. *cucumerium* (control) exhibited serious disease symptom (Figure 5A). Compared to the control, the seedlings treated with *P. polymyxa* WLY78 did not show disease symptoms and the seedlings treated with the *fusA^−^* mutant exhibited slight disease symptoms. The cucumber seedlings at 21 days of control exhibited very serious disease symptoms, while the seedlings treated with *P. polymyxa* WLY78 showed nearly no disease symptoms and the seedlings treated with the *fusA^−^* mutant exhibited serious disease symptoms. These results suggest that *P. polymyxa* WLY78 effectively controlled cucumber wilt disease, while the mutation in the *fus* gene cluster greatly decreased the biological control efficacy of *P. polymyxa* WLY78. The biological control efficacy of *P. polymyxa* WLY78 and the *fusA^−^* mutant on cucumber wilt disease were 73% and 34%, respectively (Figure 5B). Furthermore, the rhizosphere soil without treatment of *P. polymyxa* strains showed high quantities (~3.0×10^3^ CFU/g soil) of *F. oxysporum* f. sp. *cucumerium*, while the quantities of *F. oxysporum* f. sp. *cucumerium* in the rhizosphere soil treated with *P. polymyxa* WLY78 and the *fusA^−^* mutant were decreased to 0.6 × 10^3^ CFU/g of soil and 2.33 × 10^3^ CFU/g of soil, respectively (Figure 5C). These results indicate that fusaricidin plays an important role in suppressing Fusarium wilt of cucumber.

### 2.6. Fusaricidin Is Involved in Inducing Systemic Resistance

**Figure 6:**
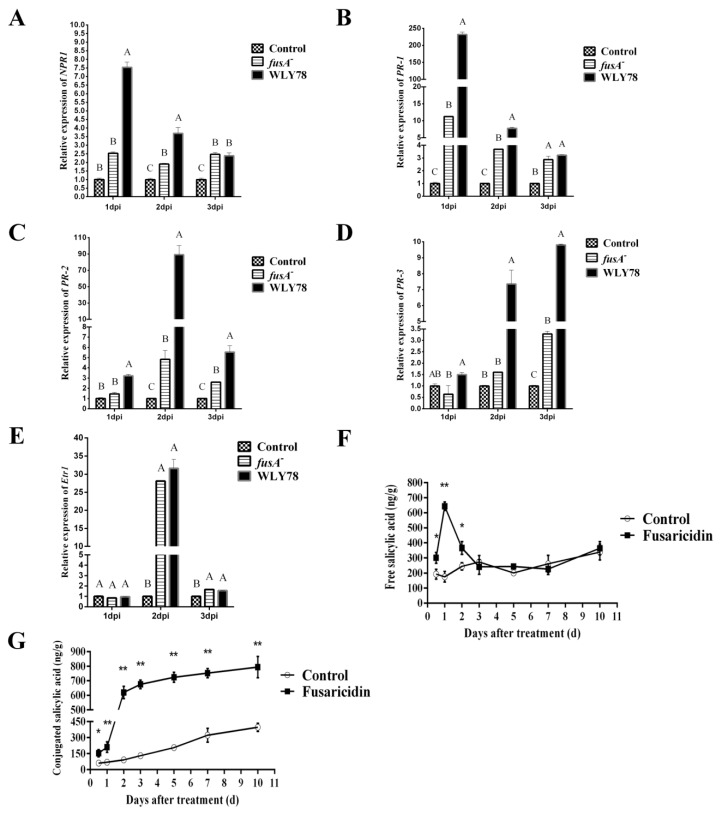
Relative expression levels of genes *NPR1* (**A**), *Etr1* (**B**), *PR1* (**C**), *PR2* (**D**), and *PR3* (**E**) in leaves of *P. polymyxa* WLY78, the *fusA*^−^ mutant, and water treatment at days 1, 2. and 3. Accumulation of free salicylic acid (**F**) and conjugated salicylic acid (**G**) in roots was induced by fusaricidin. Different letters or asterisks indicate significant differences at *p* < 0.01, according to the Duncan multiple range test. Error bars indicate standard deviations among triplicates.

As above described, *P. polymyxa* WLY78 was effective in suppressing Fusarium wilt of cucumber over 20 days. In light of this long-term effect, we hypothesized that systemic plant resistance might take place when *P. polymyxa* WLY78 was applied. Meanwhile, facing this microbial challenge, the plant cells have to make a rapid decision to prevent infection. Therefore, the expression levels of *NPR1* (for regulate protein non-expressor of pathogenesis-related genes 1), *PR1* (for pathogenesis-related protein 1), *PR2* (for β-1,3-glucanase), *PR3* (for chitinase), and *Etr1* (for ethylene receptor) in cucumber leaves at days 1, 2, and 3 post inoculation were comparatively analyzed by qRT-PCR. As shown in Figure 6A, the expression of *NPR1* was enhanced in *P. polymyxa* WLY78 treatment at day 1, which was significantly higher than that of the *fusA^−^* treatment and control. Then, the expression levels decreased at day 3 and showed no significant changes in response to WLY78 when compared with *fusA^−^* treatment. Similarly, the expression levels of the *PR1* gene in *P. polymyxa* WLY78 treatment at day 1 was 20-fold higher than that of the *fusA^−^* treatment and then decreased to the same levels as the *fusA^−^* treatment did at day 3 (Figure 6B). The expression levels of *PR2* at days 1, 2, and 3 in *P. polymyxa* WLY78 treatment was 1-fold, 17-fold, and 1-fold higher than that of the *fusA^−^* treatment, respectively (Figure 6C). The expression levels of *PR3* in *P. polymyxa* WLY78 treatment at days 1, 2, and 3 was 2-fold, 3-fold, and 2-fold higher than those in the *fusA^−^* treatment group, respectively (Figure 6D). In contrast, the expression levels of *Etr1* were similar in the *fusA^−^* treatment and *P. polymyxa* WLY78 treatment (Figure 6E). These results indicate that fusaricidin produced by *P. polymyxa* WLY78 could induce systemic resistance through the SA pathway, and not dependent on the ethylene pathway. To further support our findings, we determined the free SA and conjugated SA concentration in cucumber roots by treatment with purified fusaricidins and found that this systemic resistance was dependent on the SA signal. In Figure 6F, the free SA concentration significantly accumulated in roots at day 0.5, then reached a peak at day 1 and decreased to normal level at day 2 after treatment, suggesting that the free SA might be transformed into conjugated forms or transported to neighbor cells. Indeed, at day 2, an obvious increment of the conjugated SA concentration was detected in fusaricidin treatment, demonstrating that the free SA was transformed into conjugated SA (Figure 6G). In general, the fusaricidin treatment could elicit the SA accumulation in roots and then induce systemic resistance to protect the cucumber seedlings. 

## 3. Discussion

Antibiotics produced by *P. polymyxa* strains play important roles in controlling various plant diseases [33,34]. In this study, *P. polymyxa* WLY78 had inhibitory activities against various plant pathogenic fungi: *F. oxysporum* f. sp. *cucumerium*, *F. asiaticum, F. monilifrome, V. ablo-atrum*, *F. graminearum*, *M. persoon*, *A. mali*, *B. cinereal*, and *A. niger*. Genome analysis showed that there were eight biosynthesis gene clusters (*fus*, *pab*, *pmx*, *pbt*, *tri*, *dhb*, *pade,* and *paen*) that might be involved in synthesizing potential antibiotics for its wide-spectrum inhibitory character (Figure 1A,C). Among the eight gene clusters, only five gene clusters (*fus*, *pab*, *pmx*, *tri*, and *paen*) from WLY78 showed high similarities (>60%) with those known antibiotic biosynthetic clusters. Although the five highly potential antibiotic biosynthetic gene clusters showed a more or less inhibitory effect against fungi or bacteria, in fact, according to the literature, the *fus* gene cluster is involved in synthesizing fusaricidin, which is able to inhibit pathogenic fungi including *A. niger*, *Leptosphaeria maculans*, and *Penicillium expansum* [5,7,35]. The *pab* gene cluster is involved in synthesizing paenicidin, which has been reported to inhibit *Campylobacter jejuni* [36]; the *pmx* and *tri* gene clusters are involved in synthesizing polymyxin and tridecaptin, respectively, which are strongly against Gram-negative bacteria [37,38]; the *paen* gene cluster is responsible for synthesizing paenibacillin, which can effectively inhibit Gram-positive bacteria [39]. Furthermore, the other three gene clusters (*pbt*, *dhb*, and *pade*) showed low similarities (<60%) with the corresponding reported antibiotic biosynthetic gene clusters, indicating their functions are incomplete. Therefore, the *pbt*, *dhb*, and *pade* gene cluster may fail to synthesize paenibacterin, bacillibactin, and paeninodin, respectively, as previously reported to inhibit fungi [39,40,41]. Furthermore, by disrupting each core structural gene of the eight antibiotic biosynthesis gene clusters in our strain of *P. polymyxa* WLY78, we found that the *fus* gene cluster might play a crucial role in inhibiting various fungi (Figure 1B,C). Therefore, our results strongly support that fusaricidin is the major antifungal compound in *P. polymyxa* [42,43,44]. 

Further mutation analysis demonstrated that the seven genes (*fusG*, *fusF*, *fusE*, *fusD*, *fusC*, *fusB*, and *fusA*) within the *fus* gene cluster were essential for antifungal activity, but *fusTE* was not (Figure 2B). In addition, our analysis by RT-PCR showed that the seven genes (*fusG*, *fusF*, *fusE*, *fusD*, *fusC*, *fusB*, and *fusA*) were organized as an operon, and *fusTE* was independently transcribed (Figure 2A). Moreover, the HPLC results and purified fractions of each mutant confirmed that the *fusTE* was not necessary in producing fusaricidins (Figure 2C,D). Our results are different from the suggestion in a previous report that the entire *fus* cluster could consist of eight genes (*fusG*, *fusF*, *fusE*, *fusD*, *fusC*, *fusB*, *fusA,* and *fusTE*) [45,46]. Since the products of *fusG*, *fusF*, *fusE*, *fusD*, *fusc*, and *fusB* are crucial for synthesizing the lipid moiety of fusaricidin (Table 1), our findings also support the finding that the lipid moiety of fusaricidin is crucial for its antibacterial effect. Previously, a model was proposed to explain the interaction of the most antimicrobial lipopeptide with the membrane [47]. The outmost phospholipid of the bacterial membranes is surface exposed and carry a negative charge. In contrast, fusaricidin consists of a positive charge lipid moiety and six amphiphilic amino acids, which might contribute greatly to its interaction with membranes. However, there is increasing evidence to indicate that antibacterial peptides have intracellular targets such as by inhibiting cell–wall synthesis and binding to DNA [48]. In our study, we observed that WLY78 could inhibit spore germination and hyphae growth. Furthermore, this inhibiting action is due to fusaricidins, which cause cytoplasm leakage such as intracellular nucleic acid and proteins (Figure 4D). Our results are consistent with reports that the main mode of fusaricidin against bacteria is due to the disruption of membrane ion transport systems [20].

A group of fusaricidins were reported to be uniquely produced by *P. polymyxa* strains. For example, *P. polymyxa* E681 produced a mixture of two kinds of fusaricidins, LI-F05b (911 Da) and LI-F08b (925 Da) [43]. Interestingly, Kuroda et al. used the same *P. polymyxa* strain E681 to produce five kinds of fusaricidins by using LC-MS [17]. Mass spectrometry fragment ion analysis is a novel and powerful method, particularly for the structural characterization of natural compounds. Through these means to elucidate the structure, more than 10 novel fusaricidins were characterized in *P. polymyxa* M1 [49]. Additionally, two hitherto unknown fusaricidins were obtained from fermentation broths of *Paenibacillus* sp. strain Lu16774 [50]. Li et al. demonstrated that *P. polymyxa* SQR-21 could also produce four kinds of fusaricidins, A (883 Da), B (897 Da), C (947 Da), and D (961 Da) [46]. Using a similar method, we demonstrated that *P. polymyxa* WLY78 produced seven forms of fusaricidins and all of these variants were characterized efficiently by LC-MS-MS fragment ion analysis (Figure 3A–H). Our and other results revealed that the fusaricidin produced by *P. polymyxa* strains shows a much higher complexity than expected. Furthermore, the fusaricidins were reported to have a low hemolytic activity and cytotoxicity for mice [15]. These characteristics make *P. polymyxa* strains likely to be widely applied in agriculture.

One important criterion for biological control agents is their control efficacy. However, the role of fusaricidin in suppressing pathogens is rarely performed in vivo and is performed, in most cases, in vitro by assaying the antifungal activity [43,44]. In this study, we demonstrated that *P. polymyxa* WLY78 suppressed the Fusarium wilt of cucumber more effectively than the fusaricidin-deficient mutant (Figure 6), suggesting that fusaricidin played an important role in suppressing Fusarium wilt of cucumber in vivo. Furthermore, we found that the systemic resistance was elicited, since the NPR1, an important regulator of SAR and ISR, was induced by WLY78 (Figure 6A). Moreover, the resistance induced by WLY78 is dependent on the SA-mediated pathway, but not on the ethylene-mediated pathway (Figure 6B–E). Additionally, those PR proteins expressed rapidly and strongly in the first three days, which allowed the plants to immediately respond to further pathogenic invasion. Although the lasting time of PR-1 was short, there were still other PR proteins such as PR-2 and PR-3. These PR proteins might respond to SA at different times and protect plants in different ways.

Although systemic resistance induced by beneficial bacteria is often regulated through SA independent pathways, several PGPRs have been reported to trigger the SA dependent type of resistance such as *Paenibacillus alvei* K165 and *Pseudomonas fluorescens* SS101 [51,52]. Similarly, our analyses further showed significantly higher free SA concentrations in the roots of fusaricidin-treated plants, demonstrating the fusaricidin produced by WLY78 could induce systemic resistance via the SA pathway. As the SA signal triggered by beneficial rhizobacteria WLY78 is likely to follow the SAR signaling pathway, we prefer it to be SA-mediated systemic resistance.

The excessive free SA would convert to conjugated SA as storage, which could be hydrolyzed to release SA in the extracellular spaces when necessary [53]. Similar changes of free SA and conjugated SA were also determined in our study (Figure 6F,G). In all, our study for the first time demonstrates that the fusaricidin compounds produced by *P. polymyxa* WLY78 are able to elicit systemic resistance via the SA signal against Fusarium wilt of cucumber.

**Figure 7:**
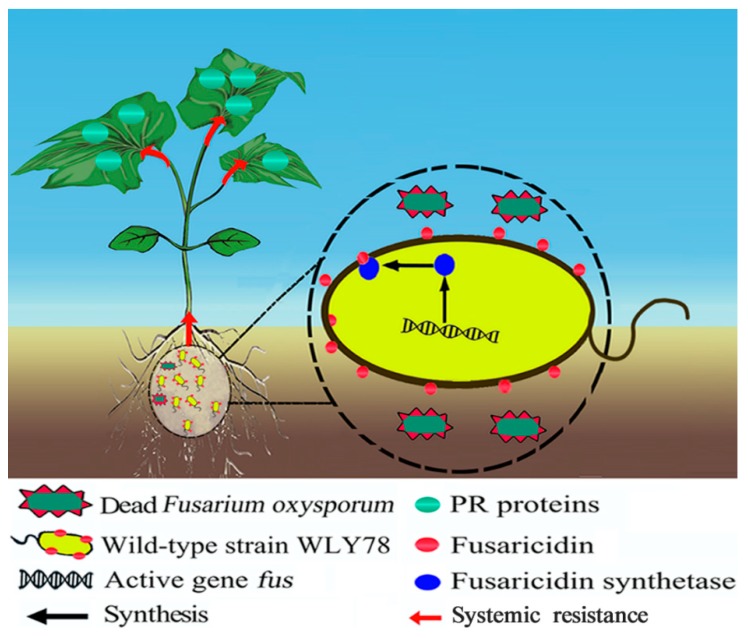
The proposed model of *P. polymyxa* WLY78 suppresses Fusarium wilt of cucumber. *P. polymyxa* WLY78 produces fusaricidins to inhibit *F. oxysporum* f. sp. *cucumerium* directly and elicit plant systemic resistance against Fusarium wilt of cucumber.

Based on current data and previous studies, we proposed a mode of the fusaricidin of *P. polymyxa* WLY78 in suppressing Fusarium wilt of cucumber (Figure 7). *P. polymyxa* WLY78 produces fusaricidins, and then fusaricidins directly inhibit spore germination and disrupts the hyphal tips of *F. oxysporum* f. sp. *cucumerium* in the rhizosphere of cucumber. Additionally, fusaricidins elicit plant systemic resistance via the SA signal against Fusarium wilt of cucumber.

## 4. Materials and Methods

### 4.1. Microorganisms, Plasmids, and Culture Conditions

The source of strains and plasmids are listed in Table S1. *Escherichia coli* DH5α was cultivated at 37 °C in Luria-Bertani (LB) medium for the cloning of plasmids. *P. polymyxa* strains were cultivated at 30 °C in LB medium, and Katznelson & Lochhead (KL) broth for the production of fusaricidin [54]. For the nitrogenase activity assays, *P. polymyxa* strains were grown in nitrogen-limited medium under anaerobic condition [31]. When necessary, the antibiotics were added to the medium at the following concentrations: 100 μg/mL ampicillin or 5 μg/mL erythromycin. The pathogenic fungi (*F. oxysporum* f. sp. *cucumerium*, *F. asiaticum*, *F. moniliforme*, *F. graminearum*, *Verticillium albo-atrum*, *Monilia persoon*, *Alternaria mali*, *Botrytis cinereal*, and *Aspergillus niger*) were cultivated at 28 °C in potato dextrose agar (PDA) medium.

### 4.2. Disruption of the Gene Cluster Involved in Synthesis of the Potential Antifungal Substances

The gene clusters that might be involved in the synthesis of antifungal substances in the genome of *P. polymyxa* WLY78 (GenBank: ALJV00000000) were predicted by antiSMASH (https://antismash.secondarymetabolites.org/#!/start). There are eight gene clusters that might be involved in the synthesis of the potential antibiotics in this bacterium and each gene cluster contained one to four core genes with lengths of 4–23 kb. 

To investigate whether the eight gene clusters were involved in antibiotic production, each gene cluster was disrupted by deleting a partial (2–4 kb) coding region of a core gene via homologous recombination. Two homologous arms (each ~1 kb in length), flanking the deleted coding region of a core gene, were amplified from the genomic DNA of *P. polymyxa* WLY78. Then, the two homologous arms were assembled into the suicide plasmid pRN5101 digested by *Bam*HI (New England Biolabs Inc., Ipswich, MA, USA), yielding a recombinant plasmid, before the plasmid was transformed into *P. polymyxa* WLY78 as described [55]. For example, the *fus* gene cluster was disrupted by deleting a 3.7 kb region of the *fusA* (core gene) by homologous recombination. Briefly, a 5’ homologous arm (1 kb) flanking the upstream of the coding region of *fusA* was amplified using primers fusAUf and fusAUr, and a 3’ homologous arm (1 kb) flanking the downstream of the coding region of *fusA* was amplified using primers fusADf and fusADr. The two homologous arms were assembled into the suicide plasmid pRN5101 digested by *Bam*HI (New England Biolabs Inc.), yielding a recombinant plasmid pRN5101-TFfusA, and then the plasmid was transformed into *P. polymyxa* WLY78. The single-crossover transformants were selected for erythromycin resistance. Subsequently, the double-crossover transformants were selected from the initial erythromycin resistance transformants after several rounds of non-selective growth at 39 °C. The double-crossover mutants were identified by PCR and then their antifungal activities against *F. oxysporum* f. sp. *cucumerium* were examined. The primers for PCR are listed in Supplementary Table S2. The nucleotide sequences of those genes which were disrupted were deposited in GenBank (*fusA* AYC81015, *pabB* MN087474, *pmxA1* MN087480, *pbtC* MN087479, *triE* MN087478, *dhbE* MN087477, *padeC* MN087476, and *paenC* MN087475).

### 4.3. Mutation of Each Gene within the fus Gene Cluster

The nucleotide sequence of the *fus* gene cluster was deposited in GenBank (accession number MH368541.1). There were eight genes (*fusA, fusB, fusC, fusD*, *fusE*, *fusF*, *fusG*, and *fusTE*) within the *fus* gene cluster predicted by using antiSMASH. Each gene within the *fus* gene cluster was mutated by deleting its coding region via homologous recombination as described above. The primers for PCR are listed in Supplementary Table S3.

### 4.4. Antifungal Activity Assay

*P. polymyxa* WLY78 and its mutants were grown in LB broth, and the bacterial cells were collected after 12 h of cultivation at 30 °C. The bacterial cells were suspended in sterilized water with a concentration of 10^7^ colony-forming unit (CFU)/mL. To compare the antifungal activities of *P. polymyxa* WLY78 with that of the mutants, *F. oxysporum* f. sp. *cucumerium* was inoculated onto the center of the plate containing the PDA medium. Then, individual 0.5 μL of cell suspensions from *P. polymyxa* WLY78 and from mutant strains were inoculated onto the two sides of *F. oxysporum* f. sp. *cucumerium* at the same distance of 2.5 cm. All plates were cultured at 28 °C for four days. Then, the inhibition effect of fungal growth was recorded. 

### 4.5. RT-PCR Analysis

To verify if the eight genes within the *fus* cluster were organized in a single operon, several primers designed to span across intergenic regions were used to conduct the RT-PCR reaction. Total RNA of the *P. polymyxa* WLY78 cells collected from the LB broth was extracted by RNAiso Plus and converted into cDNA by a Reverse Transcription Reagent Kit (TaKaRa, Kusatsu Shiga, Japan). The primers for RT-PCR are listed in Table S4.

### 4.6. Extraction, Purification, and Antifungal Activity Assay of Fusaricidin

To extract the purified fusaricidin, the cells of *P. polymyxa* WLY78 and the *fus* mutants were grown in 100 mL of KL broth at 30 °C for 72 h with shaking at 200 rpm. The cells were collected by centrifugation at 12,000 rpm, 4 °C for 5 min, and extracted with 5 mL of methanol for 12 h. Then, the crude extracts were centrifuged at 12,000 rpm for 5 min to remove the cells and condensed by a vacuum freeze dryer, until ~2 mL of methanol remained. The crude extracts were fractioned by HPLC (Shimadzu LC-20AP) using the following method. A total of 1.5 mL of the methanol extract was injected into a C18 reversed-phase column (250 mm × 20 mm) with 90% acetonitrile (*v*/*v*) in 0.1% trifluoroacetic acid solution at a flow rate of 20 mL/min and detected by UV at 210 nm. The purified fractions were collected, air dried, and dissolved in 1 mL of methanol for antifungal activity assay. Then, 250 μL extracts of the purified fractions were dropped into the sterile iron rings (0.7 cm in diameter) on the PDA medium that had been mixed with 1 mL of the *F. oxysporum* f. sp. *cucumerium* spores (10^6^ CFU/mL). After cultivation at 28 °C for four days, the size of the inhibition zone was recorded.

### 4.7. Identification of Fusaricidin

The active HPLC fractions were analyzed by LC-MS (Agilent 6520) under positive mode with an electrospray ionization/collision induced dissociation source. The following conditions were applied: gas temperature 300 °C, drying gas 5 L/min, nebulizer 30 psig, capillary voltage at 3.5 kV, spray voltage was 4 kV.

### 4.8. Acetylene Reduction Assays of Nitrogenase Activity

The nitrogenase activity of *P. polymyxa* WLY78 and the *fusA^−^* mutant was determined by using acetylene reduction assays as described [55]. The nitrogenase activity was expressed in nmol C_2_H_4_/mg protein/h.

### 4.9. Inhibitory Mode of Fusaricidin against F. oxysporum f. sp. cucumerium

To investigate the inhibitory mode of fusaricidin against *F. oxysporum* f. sp. *cucumerium*, 10 μL of the methanol extracts from *P. polymyxa* WLY78 and the *fusA^−^* mutant and 10 μL of the fusaricidin extracts were respectively added into 0.5 mL suspensions of the mycelia or spores of *F. oxysporum* f. sp. *cucumerium*. A total of 10 μL of methanol was used as the controls. For preparation of the spore suspension (10^6^ CFU/mL), the spores were brushed in sterile PDA liquid broth from the disk. For preparation of the mycelia suspension, 1 mL of the spore suspension (10^6^ CFU/mL) above was inoculated into 5 mL of PDA liquid broth and cultured for six hours at 28 °C with 200 rpm. The growth status of the fungal mycelia and fungal spores in the suspensions was observed under microscope after incubation at 28 °C with 200 rpm.

A total of 1 mL of spore suspension (10^6^ CFU/mL) was treated with 10 μL of fusaricidin extracts at 28 °C. A total of 10 μL of methanol was used as a control. After treatment for six hours, the supernatant was obtained by centrifugation at 5000 rpm for 2 min. Then, the OD_260_ and OD_280_ values were detected to evaluate the leakage of nucleic acid and proteins from *F. oxysporum* f. sp. *cucumerium*.

### 4.10. Plant Growth Conditions

Cucumber (*Cucumis sativus* Linn. variety Zhongnong 16) seeds were surface-sterilized as described previously [56]. The seedlings used for evaluating the control efficacy and the quantity of rhizosphere fungi were planted in the mixture containing 100 g peat soil, 200 g vermiculite, and 100 mL of *F. oxysporum* f. sp. *cucumerium* spore suspensions (10^4^ CFU/mL) in 15-cm pots. For detecting defense-related gene expression and SA accumulation, the seedlings were planted in 250-mL Erlenmeyer flasks containing 20 mL of Murashige & Skoog solid medium [57]. All cucumber seedlings were cultivated in a growth chamber with 16 h day (27 °C, 10,000 lux) and 8 h night (25 °C, 0 lux) at 70% relative humidity.

### 4.11. Biocontrol Efficacy Assay

To confirm the correlation between fusaricidin production and the biological control efficacy, we compared the biological control efficacy of *P. polymyxa* WLY78 with that of the *fusA*^−^ mutant against Fusarium wilt of cucumber. The cucumber seedlings were planted in pots as described above. Individual 100 mL of cells suspensions (10^4^ CFU/mL) of *P. polymyxa* WLY78 and the *fusA*^−^ mutant (10^4^ CFU/mL) were respectively inoculated into the rhizosphere of seedlings, with 100 mL of water being used for the control group. Each group contained fifteen seedlings. Then, the disease severity was investigated at day 21 post inoculation using the following grades (g): 0, plants with no yellowing or wilting symptoms; 1, plants with <25% of leaves with yellow spots; 2, plants with 25~50% of leaves with yellow spots; 3, plants with 50~75% of leaves with yellow spots; 4, plants with 75~100% of leaves with yellow spots; and 5, plants with 100% of leaves with yellow spots [58]. The disease severity (DS) was determined by the following formula:

$$\mathrm{DS}=\frac{\sum \left(gN_{g}\right)}{hN_{t}}$$

where *g* is the grade value; *N_g_* is the number of plants of the corresponding grade; *h* is the highest grade; and *N_t_* is the total number of plants in each group.

Then, the biological control efficacy (BE) was determined by the following formula:

$$\mathrm{BE}\left(\%\right)=\left[1-\frac{{\mathrm{DS}}_{treatment}}{{\mathrm{DS}}_{control}}\right]\times 100\%$$

The quantities of *F. oxysporum* f. sp. *cucumerium* in the cucumber rhizosphere soil were detected as described previously [59]. Briefly, 5 g of seedling rhizosphere soil from *P. polymyxa* WLY78 treatment, the *fusA^−^* treatment, and water (control) treatment was respectively sampled at day 21 post inoculation. The sampled soil was suspended in 50 mL of 0.9% NaCl solution and then the suspension was spread on fungi selective medium. The colonies were counted after incubation for three days at 25 °C. 

### 4.12. Detection of Expression Levels of Plant Resistance Gene

To confirm whether the plant resistance in cucumber seedlings was induced by fusaricidin, we compared the relative expression level of plant resistance genes (*NPR1*, *PR1*, *PR2*, *PR3*, and *Etr1*) in cucumber leaves between *P. polymyxa* WLY78 treatment and the *fusA*^−^ treatment by the following method. At the trefoil stage, the cell suspensions (3 mL, 10^4^ CFU/mL) of *P. polymyxa* WLY78 and the *fusA*^−^ mutant were respectively inoculated into the rhizosphere medium of seedlings. A sample of 3 mL of water was inoculated as a control. The first leaves were sampled at days 1, 2, and 3 after treatment. The total RNA of sampled leaves was extracted by an RNAiso Plus Kit and converted into cDNA by a Reverse Transcription Reagent Kit (TaKaRa). The qRT-PCR program was conducted as described previously [56]. Primers used for qRT-PCR are listed in Supplementary Table S5. The relative expression level was calculated using the ΔΔ*C*t method [60].

### 4.13. Detection of Salicylic Acid

At the trefoil stage, the purified fusaricidin obtained above were dissolved in 3 mL of water and was then inoculated into the rhizosphere of cucumber seedlings, with 3 mL of water used for the control group. The roots (0.5 g) of the cucumber seedlings were collected at days 0.5, 1, 2, 3, 5, 7, 10 after treatment. The SA was extracted as described with minor modifications [61]. The samples were ground in liquid N_2_ and extracted in 1 mL of 80% cold ethanol, followed by centrifugation at 4 °C with 12,000 rpm for 2 min and then the precipitates were extracted again in 0.5 mL of ethanol. The combined ethanol extracts were dried by vacuum freeze dryer, until ~0.2 mL of the water remained, then extracted twice with 0.5 mL of ethyl acetate. Meanwhile, 0.4 mL of HCl (6 mol/L) was mixed into the water phase at 80 °C for 1 h, and then extracted twice with 0.5 mL of ethyl acetate. Both of the two ethyl acetate phases were dried, and the dried material was re-suspended in 0.2 mL of methanol to obtain free SA and conjugated SA, respectively. The suspension was centrifuged at 12,000 rpm for 2 min and filtered with a 0.22 μm filter. SA was detected at 300 nm by a HPLC (Shimadzu LC-20AT) equipped with a UV detector. A total of 20 μL of sample was injected into a C-18 reverse-phase column (4.6 × 150 mm) with 80% methanol (*v*/*v*) in 0.1% acetic acid solution at a flow rate of 0.8 mL/min. 

### 4.14. Statistical Analysis

All of the experiments were repeated three times with a similar result. The significant difference (*p* ˂ 0.01) of data was analyzed by one-way ANOVA with Duncan’s multiple-range test using SPSS version 22 statistical software (SPSS, Chicago, IL, USA).

## Figures and Tables

**Table 1:** Name, length, and predicted function of genes within the *fus* gene cluster of *P. polymyxa* WLY78.

Gene	Length (bp)	Accession NO. of GenBank	Predicted Product
*fusTE*	1035	AYC81014.1	Alpa/beta hydrolase-thioesterase
*fusG*	771	AYC81021.1	Enoyl-acyl carrier protein reductase
*fusF*	1461	AYC81020.1	Acyl-CoA ligase
*fusE*	1224	AYC81019.1	Aldehyde dehydrogenase
*fusD*	1701	AYC81018.1	Acetolactate synthase large subunit
*fusC*	1242	AYC81017.1	3-Oxoacyl-acyl carrier protein synthase
*fusB*	408	AYC81016.1	(3R)-Hydroxymyristoyl-acyl carrier protein
*fusA*	23730	AYC81015.1	Non-ribosomal polypeptide synthetase

## Supplementary Materials

Supplementary materials can be found at https://www.mdpi.com/1422-0067/20/20/5240/s1.

## Abbreviations

*fus*	Fusaricidin biosynthesis gene
*pab*	Paenicidin B biosynthesis gene
*pmx*	Polymyxin biosynthesis gene
*pbt*	Paenibacterin biosynthesis gene
*tri*	Tridecaptin biosynthesis gene
*dhb*	Bacillibactin biosynthesis gene
*pade*	Paeninodin biosynthesis gene
*paen*	Paenibacillin biosynthesis gene
LC-MS	Liquid chromatography-mass spectrum
PR	Pathogenesis-related
ISR	Induced systemic resistance
SAR	Systemic acquired resistance
SA	Salicylic acid
