# Supplementary material for: Fusaricidin Produced by Paenibacillus polymyxa WLY78 Induces Systemic Resistance against Fusarium Wilt of Cucumber
Source: Int J Mol Sci. 2019 Oct 22;20(20):5240. doi: 10.3390/ijms20205240 (PMC6829208; doi:10.3390/ijms20205240)
Supplement: Supplementary file 1 [file ijms-20-05240-s001.pdf]

Supplementary Materials: Fusaricidin Produced by *Paenibacillus polymyxa* WLY78 Induces Systemic Acquired Resistance against Fusarium wilt of Cucumber

Supplementary Tables

Table S1. Strains and plasmids.

Strains and Plasmids	Description	Source
**Bacterial Strains**		
Escherichia coli DH5α	*sup*E44Δ*lac*U169（ϕ80 *lacZ*ΔM15）*hsd*R17 *rec*A1 *end* A1 *gyr*A96 *thi*-1 *rel*A1	Solarbio
Paenibacillus zanthoxyli JH29	Wild-type	[1]
Paenibacillus beijingensis 1-18	Wild-type	[2]
Paenibacillus sabinae T27	Wild-type	[3]
Bacillus amyloliquefaciens LJ02	Wild-type	[4]
Bacillus subtilis L56	Wild-type	[5]
Paenibacillus polymyxa		
WLY78	Wild-type ACCC 03145	ACCC
fusA^{-} mutant	Partial regions of *fusA* deleted from the WLY78 genome	This work
pabB^{-} mutant	Partial region of *pabB* deleted from the WLY78 genome	This work
pmxA1^{-} mutant	Partial region of *pmxA1* deleted from the WLY78 genome WLY78gegenomgenome	This work
pbtC^{-} mutant	Partial region of *pbtC* deleted from the WLY78 genome	This work
triE^{-} mutant	Partial region of *triE* deleted from the WLY78 genome	This work
dhbE^{-} mutant	Partial region of *dhbE* deleted from the WLY78 genome	This work
padeC^{-} mutant	Partial region of *padeC* deleted from the WLY78 genome	This work
paenC^{-} mutant	Partial region of *paenC* deleted from the WLY78 genome	This work
**Fungi**		
Fusarium oxysporum f. sp. *cucumerium*	ACCC 30220	ACCC
Fusarium asiaticum	ACCC 39255	ACCC
Fusarium moniliforme	ACCC 30133	ACCC
Verticillium albo-atrum	ACCC 30053	ACCC
Fusarium graminearum	ACCC 31053	ACCC
Monilia persoon	ACCC 37407	ACCC
Alternaria mali	ACCC 30003	ACCC
Botrytis cinerea	ACCC 30387	ACCC
Aspergillus niger	ACCC 30005	ACCC
**Plasmids**		
pRN5101	A temperature-sensitive shuttle vector containing the *ori*(Ts) and *erm* of pE194ts and the *ori*Ec, *amp* and multicloning region of pBR322	[6,7]
pHY300PLK	Multiple-copy *E. coli-Bacillus* shuttle vector, *Tet*^{R}	TaKaRa
pRN5101-TFfusA	pRN5101 ligated with two flanking homologous fragments of core fusaricidin synthesis gene	This work
pRN5101-TFpabB	pRN5101 ligated with two flanking homologous fragments of core paenicidin synthesis gene	This work
pRN5101-TFpmxA1	pRN5101 ligated with two flanking homologous fragments of core polymyxin synthesis gene	This work
pRN5101-TFpbtC	pRN5101 ligated with two flanking homologous fragments of core paenibacterin synthesis gene	This work
pRN5101-TFtriE	pRN5101 ligated with two flanking homologous fragments of core tridecaptin synthesis gene	This work
pRN5101-TFdhbE	pRN5101 ligated with two flanking homologous fragments of core bacillibactin synthesis gene	This work
pRN5101-TFpadeC	pRN5101 ligated with two flanking homologous fragments of core paeninodin synthesis gene	This work
pRN5101-TFpaenC	pRN5101 ligated with two flanking homologous fragments of core paenibacillin synthesis gene	This work
pRN5101-TFfusB	pRN5101 ligated with two flanking homologous fragments of *fusB*	This work

pRN5101-TFfusC	pRN5101 ligated with two flanking homologous fragments of *fusC*	This work
pRN5101-TFfusD	pRN5101 ligated with two flanking homologous fragments of *fusD*	This work
pRN5101-TFfusE	pRN5101 ligated with two flanking homologous fragments of *fusE*	This work
pRN5101-TFfusF	pRN5101 ligated with two flanking homologous fragments of *fusF*	This work
pRN5101-TFfusG	pRN5101 ligated with two flanking homologous fragments of *fusG*	This work
pRN5101-TFfusTE	pRN5101 ligated with two flanking homologous fragments of *fusTE*	This work

Table S2. Primers for amplification of the homologous arms flanking the region of deletion in core genes.

PCR Product	Primer	Oligonucleotide Sequences (5′-3′)
5′ homologous arm flanking the region of deletion in *fusA*	fusAUf<br>fusAUr	ACGATGCGTCCGGCGTAGAGCATCCGCGCGAACGACCA<br>TTCAGGATCTGATTCCGACGCCAAGCTATTATTGG
3′ homologous arm flanking the region of deletion in *fusA*	fusADf<br>fusADr	CGTCGGAATCAGATCCTGAATTAACTGATTG<br>GCGACCACACCCGTCCTGTGATGAATGACATGCAGTTATATG
5′ homologous arm flanking the region of deletion in *pabB*	pabBUf<br>pabBUr	ACGATGCGTCCGGCGTAGAGTCCCTACATACATGATCTTATAG<br>GGAACTGACATCCATTCCCATTAAACCTC
3′ homologous arm flanking the region of deletion in *pabB*	pabBDf<br>pabBDr	TGGGAATGGATGTCAGTTCCAAAAAGAATG<br>GCGACCACACCCGTCCTGTGGGAGTATATAATTATATTGCTCTACTG
5′ homologous arm flanking the region of deletion in *pmxA1*	pmxA1Uf<br>pmxA1Ur	ACGATGCGTCCGGCGTAGAGTGGGAAGACCCAATTTAAC<br>AAGCGCACAGTATAATCGTCGCTACCGAATTTTTC
3′ homologous arm flanking the region of deletion in *pmxA1*	pmxA1Df<br>pmxA1Dr	GACGATTATACTGTGCGCTTACCTGGTC<br>GCGACCACACCCGTCCTGTGCGGGCTTTGCGCGTAAGC
5′ homologous arm flanking the region of deletion in *pbtC*	pbtCUf<br>pbtCUr	ACGATGCGTCCGGCGTAGAGATAGGCGCTTCATACTCTATTC<br>TTATGGATAACAGTTTGATTTTAACGAAAATGATG
3′ homologous arm flanking the region of deletion in *pbtC*	pbtCDf<br>pbtCDr	AATCAAACTGTTATCCATAACTTCGTCCC<br>GCGACCACACCCGTCCTGTGAATACTAAAGTAGTTAATGCCTAC
5′ homologous arm flanking the region of deletion in *triE*	triEUf<br>triEUr	ACGATGCGTCCGGCGTAGAGTCCCTCCGGTTGCGGCAA<br>CCTTTTTACCGTTATGGTAGAACATCGCAATGTCGTGC
3′ homologous arm flanking the region of deletion in *triE*	triEDf<br>triEDr	CTACCATAACGGTAAAAAGGAAATCGCATATTCATGCGGTTTG<br>GCGACCACACCCGTCCTGTGTGGAGCTAGGGGCGGCTG
5′ homologous arm flanking the region of deletion in *dhbE*	dhbEUf<br>dhbEUr	ACGATGCGTCCGGCGTAGAGCTCCACGAACGTCACCCTTG<br>GAGGAATATCCGCAAGACAGATACTCGTAATGAAAAATG
3′ homologous arm flanking the region of deletion in *dhbE*	dhbEDf<br>dhbEDr	CTGTCTTGCGGATATTCCTCCTCTTTCTGTATCGTTC<br>GCGACCACACCCGTCCTGTGCGAACTGGCACTCCGAGAATG
5′ homologous arm flanking the region of deletion in *padeC*	padeCUf<br>padeCUr	ACGATGCGTCCGGCGTAGAGAGTTAGGGACTGTATGTG<br>TCTATAGCTTTCGAATCTGTTCGGTCAG
3′ homologous arm flanking the region of deletion in *padeC*	padeCDf<br>padeCDr	ACAGATTCGAAAGCTATAGATTCCGGCAATC<br>GCGACCACACCCGTCCTGTGGCTTATCGACAAATCCCC
5′ homologous arm flanking the region of deletion in *paenC*	paenCUf<br>paenCUr	ACGATGCGTCCGGCGTAGAGAAACTGTAGTAGCACAGG<br>TACATGTACCCATCGAAAGCTTTTGCAC
3′ homologous arm flanking the region of deletion in *paenC*	paenCDf<br>paenCDr	GCTTTCGATGGGTACATGTAATTTTCCGAG<br>GCGACCACACCCGTCCTGTGCATTATGGAGATTGCATGATAAAAC

Table S3. Primers for amplification of the homologous arms flanking the genes within the *fus* cluster.

PCR Product	Primer	Oligonucleotide Sequences (5′-3′)
fusB upstream	fusBUf<br>fusBUr	ACGATGCGTCCGGCGTAGAGCGACGTATGTTCTGGCAGCAG<br>GAATAAGCCTCGGTGTGGAAGCGCACGT
fusB downstream	fusBDf<br>fusBDr	TTCCACACCGAGGCTTATTCCCCTTCCCAC<br>GCGACCACACCCGTCCTGTGGCAGGCGGTGGAGCAATC
fusC upstream	fusCUf<br>fusCUr	ACGATGCGTCCGGCGTAGAGCGGCTCATTTCCTTGAATTAACTC<br>CGTGGATACAGCCTGTGAGCGAAGAGCTAATC
fusC downstream	fusCDf<br>fusCDr	GCTCACAGGCTGTATCCACGTCCTTCGTTG<br>GCGACCACACCCGTCCTGTGGAAGAACACGAACTTTTCTGC
fusD upstream	fusDUf<br>fusDUr	ACGATGCGTCCGGCGTAGAGAACGGGGGCATGCTCCC<br>TTTGGTATGAGAAGGACGTGGATACAGTGAGAAGACG
fusD downstream	fusDDf<br>fusDDr	CACGTCCTTCTCATACCAAACTCCTCTTTTC<br>GCGACCACACCCGTCCTGTGTATGGCTGTATTCATGCC
fusE upstream	fusEUf<br>fusEUr	ACGATGCGTCCGGCGTAGAGGCCGATCATGCCATTGCTC<br>AGAATGTGCCATGACTGCGAGCTTTTTCC
fusE downstream	fusEDf	TCGCAGTCATGGCACATTCTCCCTTTCC

	fusEDr	GCGACCACACCCGTCCTGTGCGCCATGAACTACCTACC
fusF upstream	fusFUf	ACGATGCGTCCGGCGTAGAGCTGGAGGAGATACGCTTG
	fusFUr	AGGGAACGTTCAAGATATTTACCAAAACCGC
fusF downstream	fusFDf	AAATATCTTGAACGTTCCCTCCTTACATC
	fusFDr	GCGACCACACCCGTCCTGTGAAGCAACACAATTATTTTTGTG
fusG upstream	fusGUf	ACGATGCGTCCGGCGTAGAGCGACACCGTCCGTCTGCT
	fusGUr	AAAGGTGGTTTCTGGAGGGAACGTTGTGCTTATTAC
fusG downstream	fusGDf	CGTTCCCTCCAGAAACCACCTTTCTTTTTTTACATTATTAAAC
	fusGDr	GCGACCACACCCGTCCTGTGGCGGTGTTCATCACGGTG
fusTE upstream	fusTEUf	ACGATGCGTCCGGCGTAGAGCATTGCGGAAGATTCCCTAC
	fusTEUr	TATAAAGTCCTAGAAAATCTACTCCTCTATATAGCTATAATTAATC
fusTE downstream	fusTEDf	AGATTTTCTAGGACTTTATATGTTAAGGACAG
	fusTEDr	GCGACCACACCCGTCCTGTGCCATTCCCTGAAAGTATTG

Table S4. Primers for RT-PCR analysis.

PCR Product	Primer	Oligonucleotide Sequences (5′-3′)
0 intergenic spacer	0f	GAGTTGTGCCCCTTCAGCAG
	0r	GCGGTGTTCATCACGGTGA
1 intergenic spacer	1f	GTCGAGGGCTTCGAGCTTTG
	1r	GAGCTTGATGCATTGGCGG
2 intergenic spacer	2f	CACCACAATATCAGCTGGC
	2r	GTAGGCGAAGTGTGGATACG
3 intergenic spacer	3f	CAAGCGGCTTCACAAGTGAG
	3r	GTTCAACTCTGGACAGGAC
4 intergenic spacer	4f	CAGTGACCGGTCCAATTC
	4r	CTTGGAAGAAACGGCTACG
5 intergenic spacer	5f	GTACCGGTGAGGAAGCACATC
	5r	GGGTTATTGTCGGCTCTGG
6 intergenic spacer	6f	GACTTCCAACCCGTAAGC
	6r	GTTGATTGTGGAGTCGCTTGC
7 intergenic spacer	7f	CCTAAGGTCAATCCCTCCCC
	7r	GATTGTCGAGACGGTGCTGG
8 intergenic spacer	8f	GTTGCTCCGTCAGGCTTTCG
	8r	CGTCCATGTTCGCTTTCG

Table S5. Primers for qRT-PCR analysis.

Gene	Forward Primer (5′-3′)	Reverse Primer (5′-3′)	Reference
NPR1	TTACTGATAAGGGCAAGAAGGCC	AAAGTTCACAAAGAGCAGGATGG	[8]
PR1	TGCTCAACAATATGCGAACC	TCATCCACCCACAACTGAAC	[9]
PR2	GGTGACCGTCAGCGGG	TTCCAACATTACAAGCTCTAAGA	[10]
PR3	GCCTTACTCCATAACATCACTCC	GATTTCGATATCGAGTCTGGCT	[10]
ETR1	GCCATTGTTGCAAAAGCAGA	GCCAAAGACCACTGCCACA	[11]
EF1α	ACTGTGCTGTCCTCATTATTG	AGGGTGAAAGCAAGAAGAGC	[12]

Table S6. Physiological and biochemical characteristics of *P. polymyxa* WLY78 and *fusA*⁻ mutant.

Physiological and Biochemical Index	WLY78	*fusA*⁻ Mutant
Catalase activity	+	+
Nitrate reduction	+	+
H_2S produced	-	-
V. *Paenibacillus* Test	+	+
Growth in NaCl range at (%, w/v)	0-4%	0-4%
Indole production	-	-
Egg yolk reaction	-	-
Anaerobic growth	-	-
Oxidase activity	-	-
Temperature for growth range	15-42℃	15-42℃
Motility	+	+
**Hydrolysis of**		
Gelatin	+	+
Aesculin	+	+

Casein	+	+
Tyrosine	-	-
Urea	-	-
Cellulose	+	+
Tween 20	-	-
Tween 80	-	-
**Utilization of sole carbon source**		
Starch	+	+
Inositol	-	-
l-Arabinose	+	+
d-Trehalose	+	+
Glycerol	+	+
Ascorbic acid	+	+
Proline	+	+
Cystine	-	-
Threonine	-	-
Valine	-	-
Arginine	+	+
Citric acid	-	-
Sucrose	+	+
Xylose	+	+
Maltose	+	+
Tyrosine	-	-
Mannitol	+	+
Glucose	+	+
Sorbitol	+	+
Fructose	+	+
Oxalate	-	-
Galactose	+	+
Ribose	+	+
Glycogen	+	+
Rhamnose	-	-
Lysine	+	+

"+" means positive; "—" means negative.

Table S7. Summary of genes from the paenicidin B biosynthetic gene cluster.

Gene	Length (bp)	Highest BLAST Hit (% Identity)	Predicted Product
pabA	174	AHF21230.1 (69)	Paenicidin B prepropeptide
pabF	729	AHF21231.1 (89)	ABC transporter ATP binding protein
pabE	795	AHF21232.1 (67)	ABC transporter membrane-bound subunit
pabG	768	AHF21233.1 (78)	ABC transporter membrane-bound subunit
pabB	3180	AHF21234.1 (76)	Lantibiotic dehydratase
pabT	1884	AHF21235.1 (87)	Lantibiotic ABC transporter
pabC	1383	AHF21236.1 (80)	Lantibiotic cyclase

Table S8. Summary of genes from the polymyxin biosynthetic gene cluster.

Gene	Length (bp)	Highest BLAST Hit (% Identity)	Predicted Product
pmxA1	13473	ACA97576.1 (95)	Polymyxin synthetase
pmxA2	5115	ACA97576.1 (96)	Polymyxin synthetase
pmxB	3309	ACA97577.1 (96)	Polymyxin synthetase
pmxC	1827	ACA97578.1 (98)	Transporter-like protein
pmxD	1734	ACA97579.1 (99)	Transporter-like protein
pmxE	18768	ACA97580.1 (96)	Polymyxin synthetase

Table S9. Summary of genes from the paenibacterin biosynthetic gene cluster

Gene	Length (bp)	Highest BLAST Hit (% Identity)	Predicted Product
orf1	723	AJE53773.1 (100)	Thioesterase
pbtA	10110	AGM16413.1 (46)	Paenibacterin synthetase B
pbtC	17319	AGM16414.1 (47)	Paenibacterin synthetase C
orf4	8640	WP_049816874.1 (100)	Hybrid non-ribosomal peptide synthetase/type I polyketide synthase
plpA	867	AFJ14790.1 (64)	Diaminobutyrate-2-oxoglutarate aminotransferase

Table S10. Summary of genes from the tridecaptin biosynthetic gene cluster

Gene	Length (bp)	Highest BLAST Hit (% Identity)	Predicted Product
triE	11220	AHF21229.1 (91)	Tridecaptin non-ribosomal peptide synthetase
triD3	17490	AHF21228.1 (92)	Tridecaptin non-ribosomal peptide synthetase
triD2	2940	AHF21228.1 (91)	Tridecaptin non-ribosomal peptide synthetase
triD1	28113	AHF21228.1 (88)	Tridecaptin non-ribosomal peptide synthetase
triC	1875	AHF21227.1 (89)	Diaminobutyrate-2-oxoglutarate aminotransferase
TriB	1896	AHF21226.1 (87)	ABC transporter
TriA	825	AHF21225.1 (86)	Thioesterase

Table S11. Summary of genes from the bacillibactin biosynthetic gene cluster

Gene	Length (bp)	Highest BLAST Hit (% Identity)	Predicted Product
mbtH	219	CAX52685.1 (65)	Stimulator of DhbF tyrosine adenylation activity
dhbF2	2547	ABS75232.1 (98)	Amino acid adenylation domain-containing protein
dhbF1	4899	ABS75232.1 (54)	Amino acid adenylation domain-containing protein
dhbB	1053	ABS75233.1 (60)	Isochorismatase
dhbE	1656	ABS75234.1 (70)	Adenylate synthase
dhbC	1287	ABS75235.1 (52)	Isochorismate synthase
dhbA	786	ABS75236.1 (62)	2,3-Dihydro-2,3-dihydroxybenzoate Dehydrogenase

Table S12. Summary of genes from the paeninodin biosynthetic gene cluster

Gene	Length (bp)	Highest BLAST Hit (% Identity)	Predicted Product
orf1	1494	WP_069011102.1 (99)	Nucleotidyltransferase family protein
padeB2	486	WP_029516230.1 (100)	Lasso peptide biosynthesis B2
orf2	303	WP_029516229.1 (100)	Lasso peptide biosynthesis PqqD family chaperone
orf3	1044	WP_029516228.1 (99)	Serine kinase
orf4	129	WP_134902123.1 (100)	Paeninodin family lasso peptide
padeC	1926	WP_029516227.1 (99)	Asparagine synthetase B

Table S13. Summary of genes from the paenibacillin biosynthetic gene cluster

Gene	Length (bp)	Highest BLAST Hit (% Identity)	Predicted Product
paenN	759	AFS60110.1 (98)	Putative acetylase
agrC	780	AFS60109.1 (98)	Response regulator
agrA	1329	AFS60108.1 (100)	Histidine kinase
agrD	165	AFS60107.1 (100)	Auto-inducing peptide
agrB	591	AFS60106.1 (97)	Accessory gene regulator B
paenT	1782	AFS60105.1 (99)	ATP-binding cassette transporter
paenI	558	AFS60104.1 (100)	Putative immunity protein
paenC	1272	AFS60103.1 (99)	Lantibiotic cyclase
paenB	3084	AFS60102.1 (99)	Lantibiotic dehydratase
paenP	942	AFS60101.1 (100)	Peptidase
paenA	162	AFS60100.1 (100)	Paenibacillin prepropeptide

Supplementary Figures

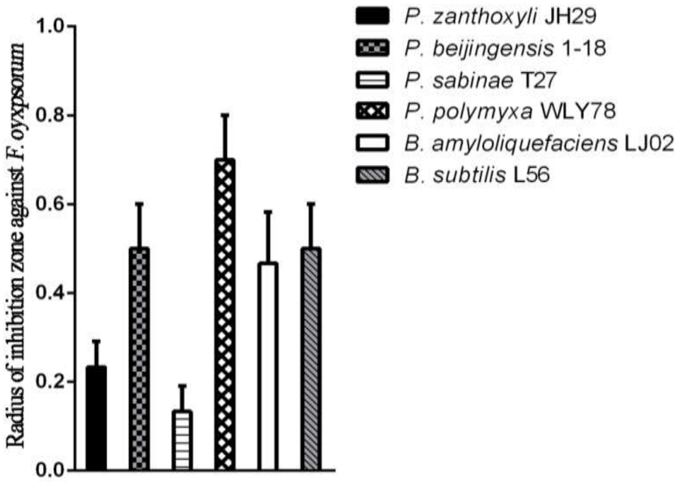

Figure 1. *P. polymyxa* WLY78 exhibits excellent antifungal activity against *F. oxysporum*. The results shown are the means ± standard deviation of a representative experiment that was repeated three times.

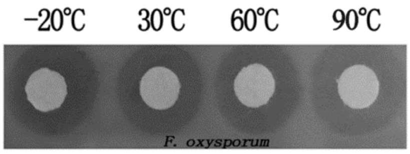

Figure 2. Methanol extracts from *P. polymyxa* WLY78 cells exhibits heat-stable antifungal activity against *F. oxysporum*.

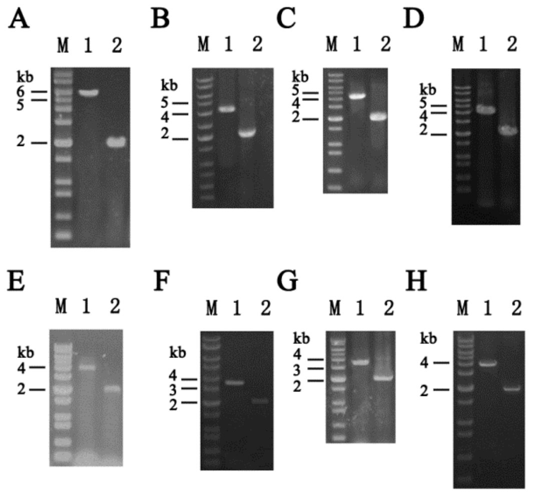

Figure 3. Agarose gel electrophoresis analysis of the disruption of genes *fusA* (A), *pabB* (B), *pmxA1* (C), *pbtC* (D), *triE* (E), *dhbE* (F), *padeC* (G) and *paenC* (H) in *P. polymyxa* WLY78. M indicates 1 kb plus DNA marker. Lane 1 indicates PCR fragments amplified by primers fusAUf/fusADr using wild-type WLY78 DNA as template and lane 2 indicates PCR fragments amplified by primers fusAUf/fusADr using *fusA*$^-$ mutant DNA as template. Compared with that amplified from wild-type WLY78, amplicon from *fusA*$^-$ mutant was ~3.7 kb smaller, confirming deletion of partial *fusA* gene (A). Lane 1 indicates PCR fragments amplified by primers pabBUf/pabBDr using wild-type WLY78 DNA as template and lane 2 indicates PCR fragments amplified by primers pabBUf/pabBDr using *pabB*$^-$ mutant DNA as template. Compared with that amplified from wild-type WLY78, amplicon from *pabB*$^-$ mutant was ~2 kb smaller, confirming deletion of partial *pabB* gene (B). Lane 1 indicates PCR fragments amplified by primers pmxA1Uf/pmxA1Dr using wild-type WLY78 DNA as template and lane 2 indicates PCR fragments amplified by primers pmxA1Uf/pmxA1Dr using *pmxA1*$^-$ mutant DNA as template. Compared with that amplified from wild-type WLY78, amplicon from *pmxA1*$^-$ mutant was 2 kb smaller, confirming deletion of partial *pmxA1* gene (C). Lane 1 indicates PCR fragments amplified by primers pbtCUf/pbtCDr using wild-type WLY78 DNA as template and lane 2 indicates PCR fragments amplified by primers pbtCUf/pbtCDr using *pbtC*$^-$ mutant DNA as template. Compared with that amplified from wild-type WLY78, amplicon from *pbtC*$^-$ mutant was ~2 kb smaller, confirming deletion of partial *pbtC* gene (D). Lane 1 indicates PCR fragments amplified by primers triEUf/triEDr using wild-type WLY78 DNA as template and lane 2 indicates PCR fragments amplified by primers triEUf/triEDr using *triE*$^-$ mutant DNA as template. Compared with that amplified from wild-type WLY78, amplicon from *triE*$^-$ mutant was ~2 kb smaller, confirming deletion of partial *triE* gene (E). Lane 1 indicates PCR fragments amplified by primers dhbEUf/dhbEDr using wild-type WLY78 DNA as template and lane 2 indicates PCR fragments amplified by primers dhbEUf/dhbEDr using *dhbE*$^-$ mutant DNA as template. Compared with that amplified from wild-type WLY78, amplicon from *dhbE*$^-$ mutant was ~1.6 kb smaller, confirming deletion of partial *dhbE* gene (F). Lane 1 indicates PCR fragments amplified by primers padeCUf/padeCDr using wild-type WLY78 DNA as template and lane 2 indicates PCR fragments amplified by primers padeCUf/padeCDr using *padeC*$^-$ mutant DNA as template. Compared with that amplified from wild-type WLY78, amplicon from *padeC*$^-$ mutant was ~1.9 kb smaller, confirming deletion of partial *padeC* gene (G). Lane 1 indicates PCR fragments amplified by primers paenCUf/paenCDr using wild-type WLY78 DNA as template and lane 2 indicates PCR fragments amplified by primers paenCUf/paenCDr using *paenC*$^-$ mutant DNA as template. Compared with that amplified from wild-type WLY78, amplicon from *paenC*$^-$ mutant was ~2 kb smaller, confirming deletion of partial *paenC* gene (H).

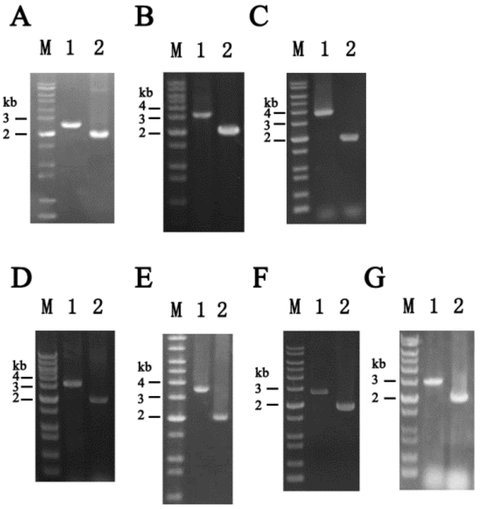

Figure 4. Agarose gel electrophoresis analysis of the deletion of genes *fusB* (A), *fusC* (B), *fusD* (C), *fusE* (D), *fusF* (E), *fusG* (F) and *fusTE* (G) in *P. polymyxa* WLY78. M indicates 1 kb plus DNA marker. Lane 1 indicates PCR fragments amplified by primers fusBUf/fusBDr using wild-type WLY78 DNA as template and lane 2 indicates PCR fragments amplified by primers fusBUf/fusBDr using *ΔfusB* mutant DNA as template. Compared with that amplified from wild-type WLY78, amplicon from *ΔfusB* mutant was ~0.4 kb smaller, confirming deletion of *fusB* gene (A). Lane 1 indicates PCR fragments amplified by primers fusCUf/fusCDr using wild-type WLY78 DNA as template and lane 2 indicates PCR fragments amplified by primers fusCUf/fusCDr using *ΔfusC* mutant DNA as template. Compared with that amplified from wild-type WLY78, amplicon from *ΔfusC* mutant was ~1.2 kb smaller, confirming deletion of *fusC* gene (B). Lane 1 indicates PCR fragments amplified by primers fusDUf/fusDDr using wild-type WLY78 DNA as template and lane 2 indicates PCR fragments amplified by primers fusDUf/fusDDr using *ΔfusD* mutant DNA as template. Compared with that amplified from wild-type WLY78, amplicon from *ΔfusD* mutant was ~1.7 kb smaller, confirming deletion of *fusD* gene (C). Lane 1 indicates PCR fragments amplified by primers fusEUf/fusEDr using wild-type WLY78 DNA as template and lane 2 indicates PCR fragments amplified by primers fusEUf/fusEDr using *ΔfusE* mutant DNA as template. Compared with that amplified from wild-type WLY78, amplicon from *ΔfusE* mutant was ~1.2 kb smaller, confirming deletion of *fusE* gene (D). Lane 1 indicates PCR fragments amplified by primers fusFUf/fusFDr using wild-type WLY78 DNA as template and lane 2 indicates PCR fragments amplified by primers fusFUf/fusFDr using *ΔfusF* mutant DNA as template. Compared with that amplified from wild-type WLY78, amplicon from *ΔfusF* mutant was ~1.4 kb smaller, confirming deletion of *fusF* gene (E). Lane 1 indicates PCR fragments amplified by primers fusGUf/fusGDr using wild-type WLY78 DNA as template and lane 2 indicates PCR fragments amplified by primers fusGUf/fusGDr using *ΔfusG* mutant DNA as template. Compared with that amplified from wild-type WLY78, amplicon from *ΔfusG* mutant was ~0.7 kb smaller, confirming deletion of *fusG* gene (F). Lane 1 indicates PCR fragments amplified by primers fusTEUf/fusTEDr using wild-type WLY78 DNA as template and lane 2 indicates PCR fragments amplified by primers fusTEUf/fusTEDr using *ΔfusTE* mutant DNA as template. Compared with that amplified from wild-type WLY78, amplicon from *ΔfusTE* mutant was ~1 kb smaller, confirming deletion of *fusTE* gene (G).

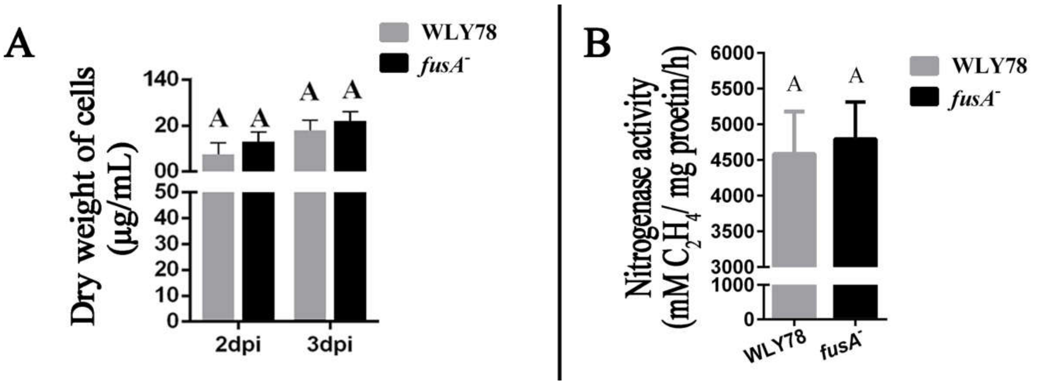

Figure 5. Dry weight of *P. polymyxa* WLY78 and the *fusA*- mutant cells collected from the KL broth (A). The nitrogenase activities of *P. polymyxa* WLY78 and the *fusA*- mutant under nitrogen-limited conditions (B). Error bars indicate standard deviation among triplicates. Different letters indicate significant difference at *P*<0.01 according to Duncan multiple range test.

Supplementary Reference

1. Ma, Y. C.; Zhang, J.; Chen, S. F., *Paenibacillus zanthoxyli* sp. nov., a novel nitrogen-fixing species isolated from the rhizosphere of *Zanthoxylum simulans*. *Int. J. Syst. Evol. Microbiol.* **2007,** 57, (4), 873-877.
2. Gao, M.; Xie, L. Q.; Wang, Y. X.; Chen, J.; Xu, J.; Zhang, X.; Sui, X. H.; Gao, J. L.; Sun, J. G., *Paenibacillus beijingensis* sp. nov., a novel nitrogen-fixing species isolated from jujube garden soil. *Antonie Van Leeuwenhoek* **2012,** 102, (4), 689-694.
3. Hong, Y.; Ma, Y.; Wu, L.; Maki, M.; Qin, W.; Chen, S., Characterization and analysis of *nifH* genes from *Paenibacillus sabinae* T27. *Microbiol. Res.* **2012,** 167, (10), 596-601.
4. Li, Y.; Gu, Y.; Li, J.; Xu, M.; Wei, Q.; Wang, Y., Biocontrol agent *Bacillus amyloliquefaciens* LJ02 induces systemic resistance against cucurbits powdery mildew. *Front. Microbiol.* **2015,** 6, 883.
5. Li, Y. B.; Shi, H. W.; Zhang, H. W.; Chen, S. F., Amerlioration of drought effects in wheat and cucumber by combined application of super absorbent polymer and poetntial biofertilizer. *PeerJ* **2018,** 7, e6073.
6. Villafane, R.; Bechhofer, D. H.; Narayanan, C. S.; Dubnau, D., Replication control genes of plasmid pE194. *J. Bacteriol.* **1987,** 169, (10), 4822-4829.
7. Lereclus, D.; Vallade, M.; Chaufaux, J.; Arantes, O.; Rambaud, S., Expansion of insecticidal host range of *Bacillus thuringiensis* by in vivo genetic recombination. *Bio/technology* **1992,** 10, (4), 418-421.
8. Xiaoming Pu; Bingyan Xie; Peiqian Li; Zhenchuan Mao; Jian Ling; Huifang Shen; Jingxin Zhang; Ning Huang; Lin., B., Analysis of the defence-related mechanism in cucumber seedlings in relation to root colonization by nonpathogenic *Fusarium oxysporum* CS-20. *FEMS Microbiol. Lett.* **2014,** 355, (2), 142-151.
9. Alizadeh, H.; Behboudi, K.; Ahmadzadeh, M.; Javan-Nikkhah, M.; Zamioudis, C.; Pieterse, C. M. J.; Bakker, P. A. H. M., Induced systemic resistance in cucumber and *Arabidopsis thaliana* by the combination of *Trichoderma harzianum* Tr6 and *Pseudomonas* sp. Ps14. *Biol. Control* **2013,** 65, (1), 14-23.
10. Sang, M. K.; Kim, K. D., Biocontrol activity and primed systemic resistance by compost water extracts against anthracnoses of pepper and cucumber. *Phytopathology* **2011,** 101, (6), 732-740.
11. Shoresh, M.; Yedidia, I.; Chet, I., Involvement of jasmonic acid/ethylene signaling pathway in the systemic resistance induced in cucumber by *Trichoderma asperellum* T203. *Phytopathology* **2005,** 95, (1), 76-84.
12. Wan, H.; Zhao, Z.; Qian, C.; Sui, Y.; Malik, A. A.; Chen, J., Selection of appropriate reference genes for gene expression studies by quantitative real-time polymerase chain reaction in cucumber. *Anal. Biochem.* **2010,** 399, (2), 257-261.
